# Segregation of Spontaneous and Training Induced Recovery from Visual Field Defects in Subacute Stroke Patients

**DOI:** 10.3389/fneur.2017.00681

**Published:** 2017-12-15

**Authors:** Douwe P. Bergsma, Joris A. Elshout, Albert V. van den Berg

**Affiliations:** ^1^Department of Cognitive Neuroscience, Section of Biophysics, Donders Centre for Neuroscience, Donders Institute for Brain, Cognition, and Behaviour, Radboud University Medical Centre, Nijmegen, Netherlands

**Keywords:** stroke, hemianopia, visual field defects, restitution training, spontaneous recovery, training-induced recovery, perimetry

## Abstract

Whether rehabilitation after stroke profits from an early start is difficult to establish as the contributions of spontaneous recovery and treatment are difficult to tease apart. Here, we use a novel training design to dissociate these components for visual rehabilitation of subacute stroke patients with visual field defects such as hemianopia. Visual discrimination training was started within 6 weeks after stroke in 17 patients. Spontaneous and training-induced recoveries were distinguished by training one-half of the defect for 8 weeks, while monitoring spontaneous recovery in the other (control) half of the defect. Next, trained and control regions were swapped, and training continued for another 8 weeks. The same paradigm was also applied to seven chronic patients for whom spontaneous recovery can be excluded and changes in the control half of the defect point to a spillover effect of training. In both groups, field stability was assessed during a no-intervention period. Defect reduction was significantly greater in the trained part of the defect than in the simultaneously untrained part of the defect irrespective of training onset (*p* = 0.001). In subacute patients, training contributed about twice as much to their defect reduction as the spontaneous recovery. Goal Attainment Scores were significantly and positively correlated with the total defect reduction (*p* = 0.01), percentage increase reading speed was significantly and positively correlated with the defect reduction induced by training (epoch 1: *p* = 0.0044; epoch 2: *p* = 0.023). Visual training adds significantly to the spontaneous recovery of visual field defects, both during training in the early and the chronic stroke phase. However, field recovery as a result of training in this subacute phase was as large as in the chronic phase. This suggests that patients benefited primarily of early onset training by gaining access to a larger visual field sooner.

## Introduction

Loss of up to one-half of the visual field (hemianopia) as result of post-chiasmatic stroke in one hemisphere occurs in about 30% of all stroke patients. Following a period of spontaneous recovery in the first 3–6 months (1, 2), the patient enters the chronic phase of hemianopia.

Rehabilitation treatment most often involves eye movement training to compensate for the visual field defect (3) rather than visual restitution training, which reduces the defect itself. The latter has long been controversial (4). However, a recent series of investigations (5–12) have argued for the more balanced view that visual training of the defect may provide an additional and valuable approach to rehabilitation of occipital stroke patients.

Brain plasticity is believed to be greater in the acute stage after stroke when there is a window for relatively quick and extensive synaptic reorganization (13). Recommendations that rehabilitation should begin “as soon as possible” or “early” are therefore common in clinical guidelines (14, 15). However, many of these recommendations are based on limited data (16), and there are no agreed definitions of what constitutes early rehabilitation (17). Thus far, visual restitution training is generally applied in the chronic phase after stroke, so that spontaneous recovery can be excluded, and changes in the visual field can be attributed to training. In this way, one can obtain an accurate estimate of the effect of the training itself (8–11). Yet, we wondered if visual restitution training would profit from an early start as suggested in the rehabilitation literature.

The effect of visual perceptual learning in normally sighted subjects is often restricted to the trained region of the visual field (18–20) and specific to the trained task (21, 22). This raises the question whether the visual recovery that is induced by visual restitution training is also limited to just the trained region and task. Several studies have shown that recovered vision after restitution training transfers to untrained visual tasks (10, 11) but only to a limited extent to untrained regions. For example, the defect reduction induced by training of the *intact* visual hemifield was significantly *smaller* than the reduction induced by training the *affected* hemifield itself, and it was not significantly different from the defect reduction following a non-intervention period (11). Because spontaneous recovery could be excluded in that study, any improvement during intact training could point to a spillover effect of training between the two hemispheres. That is, the defect reduces—albeit to limited extent—even when another part of the visual field is trained.

Following the practice of general rehabilitation medicine, one would preferably train patients in the early phase of stroke. To do so, we applied a method that builds on the observation that visual training carries over to neighboring areas only to a limited extent. That is, we used two training rounds, which targeted complementary parts of the defect [regions of interest (ROIs)], while monitoring in both training rounds the trained and the untrained half of the defect. The untrained half of the defect, which serves as an internal control for the trained half, will show spontaneous recovery and a potential spillover from the neighboring trained region. To assess that spill over, we used data from seven patients who were trained in the chronic phase of stroke using the same method. The differences between the defect reductions for the subacute phase of stroke and the chronic phase of stroke in the trained and untrained parts of the defect should allow us to distinguish between spillover, spontaneous recovery and training-induced recovery. This allows us to test the hypothesis that training in the early phase leads to a larger defect reduction than training in the chronic phase.

## Materials and Methods

The study was approved by the ethical committee CMO Arnhem–Nijmegen in correspondence with the 1964 Declaration of Helsinki.

20 Subacute stroke patients and 10 chronic stroke patients with visual field defects due to post-geniculate damage were included following written informed consent. Subacute stroke patients were screened for participation in four neurology departments of Dutch hospitals: UMC in Utrecht, St. Elisabeth Hospital in Tilburg, CWZ in Nijmegen and St. Antonius Hospital in Nieuwegein (screening; eight patients). Patients could also sign up for the study by filling out a form on our website (www.hemianopsie.nl; 12 patients), to be screened at a regional office by the first author. Chronic stroke patients all applied through the website.

Patients inclusion criteria as follows:

∗ age between 18 and 75 years;
∗ presence of homonymous visual field defect.

Patient exclusion criteria as follows:

∗ visual neglect (as assessed by line bisection test);
∗ cardiac or other implants (for the chronic patients only: MRI scans were made; to be presented elsewhere).

The intake procedure included a Goldmann perimetry measurement. Patient demographics can be found in Table S2 in Supplementary Material.

For the 30 included patients, we had to exclude the data of 3 subacute and 3 chronic patients from further analysis. In the three subacute patients, the training was not applied as intended because the defect was not divided in two equal halves (*n* = 2), or for unequal duration of the training rounds (*n* = 1). In the chronic patients, absence of an absolute defect (*n* = 1), inability to cope with training demands (*n* = 1), and anxiety for fMRI scanner measurements (*n* = 1) were reasons to exclude their data. Thus, in total, we analyzed 17 subacute and 7 chronic data sets.

The 20 subacute patients were trained by DB for this study, the 10 chronic patients were trained by JE in a parallel study using the same training paradigm.

### Study Design

Before the training, baseline values were established for visual field size (Goldmann perimetry), reading speed and Goal Attainment Scaling (GAS: personally customized and realistic goals).

**Figure 1:**
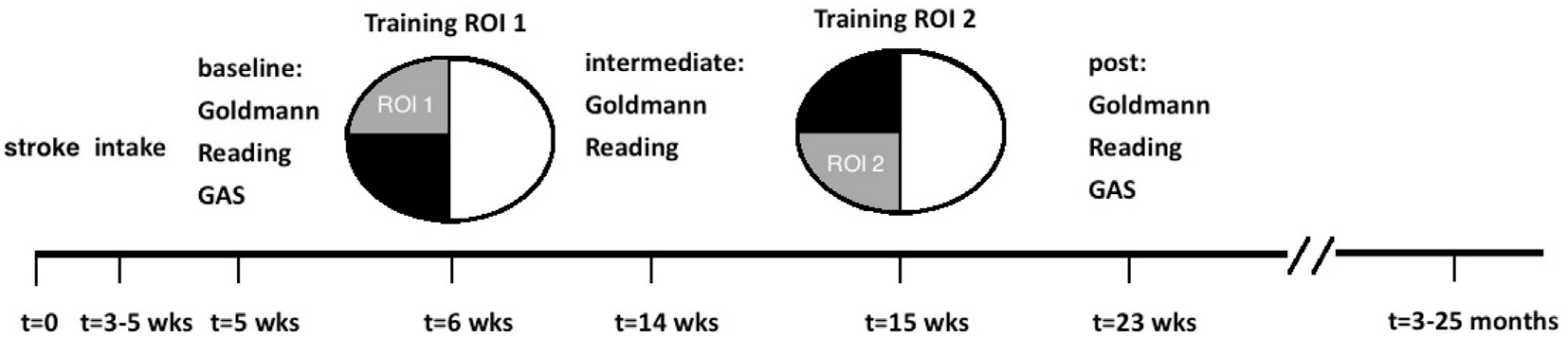
Study design and time line for subacute patients. The defect was divided into two training regions [region of interest (ROI) 1, ROI 2] of equal size. In this example, the left upper quarter field was trained first, followed by the lower left quadrant. This order was randomized between patients. For chronic patients, the study design was similar, except that the first training started at least 10 months after the stroke (and about 2 months after intake), and no follow-up measurements were taken.

Following these baseline measurements, the visual field defect was divided in equal halves using the following procedure. First, meridional angles through the defect were established that were farthest apart. Then, the average of these two outer meridional angles formed the border between the two training regions (in the case of SA15, the division was along the vertical midline). One-half of the visual field defect was trained for 8 weeks, while the other half was untrained. After this period, intermediate measurements were carried out (perimetry and reading speed tests) during the course of one week. Then, a second training period of 8 weeks was started, in which the training was applied to the other half of the defect, while the first half received no further training. Post-measurements were carried out as during baseline measurements (Figure 1). Finally, we collected follow-up perimetry data in the subacute group. The period without training, in-between the final training session and the follow-up perimetry of the subacute group, is denominated “No Intervention.”

### Training Paradigm

The training paradigm was very similar to a previous study (11). Briefly, each patient received a training unit to create a controlled training environment at home with eye fixation control. The patients trained 1 h a day, 5 days a week during both training rounds and completed at least 40 h of training per training round. Patients could freely choose when to take the required five to six daily training sessions across the day. Training hours were recorded and were continuously available to the experimenters by using the Internet for data transfer.

**Figure 2:**
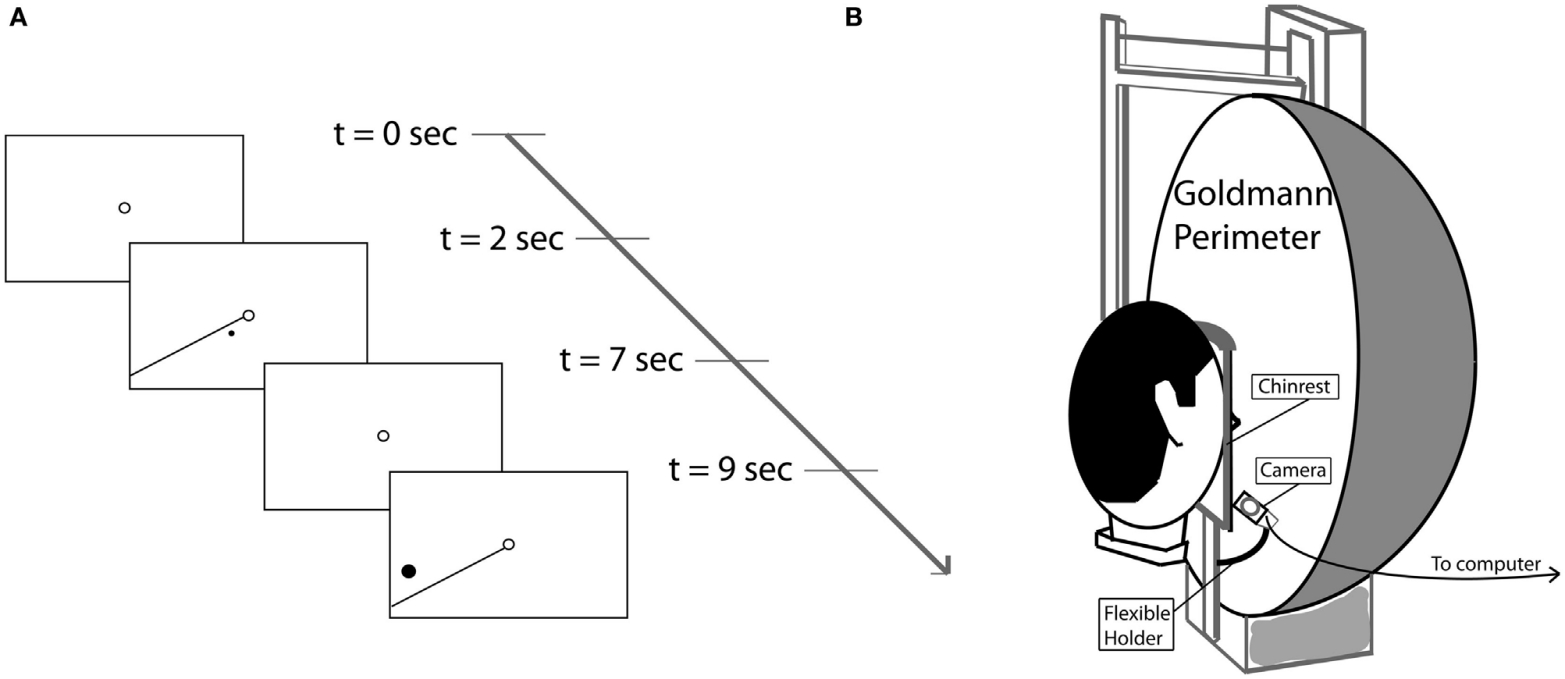
**(A)** Sequence of stimulus events during two consecutive trials. Each trial started with a single fixation point (2 s), followed by the stimulus, a target dot rotated clockwise or counterclokwise10° away from the reference line (5 s). **(B)** Goldmann perimeter with mounted Eyelink II eye tracker camera.

During training, the patient had to maintain fixation binocularly on a ring (diameter = 0.5°) at the center of the screen. High contrast stimuli (*C* > 0.9) were presented for 5 s in the border area of the region of the visual field defect that is trained. The stimuli consisted of a white dot and a simultaneously presented reference line originating in the fixation point (see Figure 2A). This line cued the approximate target location as its meridional angle differed by 10° from the training dot. If the dot *was* detected, the patient had to report (key press) whether a clockwise or counter-clockwise position was seen relative to the reference line. No response was given if no dot was detected. Dot size was at least 0.2° in diameter (at 1° eccentricity) and was scaled with eccentricity. During the intertrial interval of 2 s, only the fixation point was shown.

Duration of one training session, presenting all different stimuli once, was on average 11 min (depending on the number of trials set per session). Trials with inadequate fixation were repeated once at the end of the session, and the duration of a session would in that case be extended by the number of repetitions, with a maximum of 50% of the total number of different trials. Thus, 60–100 stimuli of a session were presented in random order in the trained ROI only.

### Perimetry

Goldmann perimetry was carried out monocularly in both eyes. For perimetry, we used *the largest and brightest* stimulus (a white Goldmann size IV stimulus with maximum luminance “4e” = 1,000 apostilb ≈ 318 cd/m^2^) against a white background with a luminance of 31.5 asb (≈10 cd/m^2^). The resulting isopter was used as boundary of the visual field. During the first measurement, the stimulus was moved from the far periphery in the affected hemifield along 8–10 meridians toward the fovea to obtain an overview into the general shape of the visual field defect. Then, a more precise perimetric map was obtained based on 20–25 trials, during which the stimulus was moved from a location deep inside the defect toward its border in a direction roughly perpendicular to that border. Stimulus speed was about 5°/s at the peripheral location and reduced to about 1°/s close to the border. Recording was stopped when the patient detected the stimulus (by tapping a pencil on the table). Fixation was continuously monitored *via* the spyglass by the experimenter and checked at random occasions using the Heijl–Krakau method for blindspot localization (23). During follow-up perimetry (description below) an eyetracker was used (Figure 2B). In both subacute and chronic patients, Goldmann perimetry was performed 1–3 days before training started, during the week in-between the two training periods and during the week after the training was stopped.

### Equivalent Cortical Surface Gain (ECSG)

Visual performance on many visual tasks strongly varies with eccentricity. The cortical magnification factor (CMF) captures this eccentricity dependence by specifying how much more mm cortex is devoted to processing 1° of visual field at the center than 1° in the periphery (24). We scaled the stimuli according to the CMF to train approximately equally large regions of cortex independent of eccentricity. Because 1° of functional recovery near the fovea corresponds to more cortical tissue than the same extent of recovery in the far periphery, we think that an estimate of the recovered cortical activation is more appropriate to characterize the training effect than the defect reduction in degrees. Thus, we express the change in eccentricity of the visual field border following training in ECSG. ECSG transforms a change of eccentricity in degrees (of Goldmann visual field isopters in the central 30° of the visual field), into an equivalent amount of functional cortex as expressed in millimeters along the retinotopic eccentricity map (10, 11). This is done using recently published fMRI data of cortical scaling in area V1 by Wu et al. (25). Each perimetric map is converted into a pixelmap, with weights (*W*) for each pixel according to its eccentricity (*E*) from the foveal projection in the map:

W(E)=21/E.

This function describes the derivative to retinal eccentricity of the inverse of the function that describes the eccentricity of the voxel’s receptive field as a function of the distance within area V1. We then summed the weighted pixel values that were located in between the defect borders of the two perimetric maps, assigning a positive sign when the pixel was located in a sector where the field defect was reduced and a negative sign when the defect was increased. Dividing the integral by π, one obtains the estimated cortical shift in the eccentricity direction (ECSG_mm) at the human primary visual cortex.

**Figure 3:**
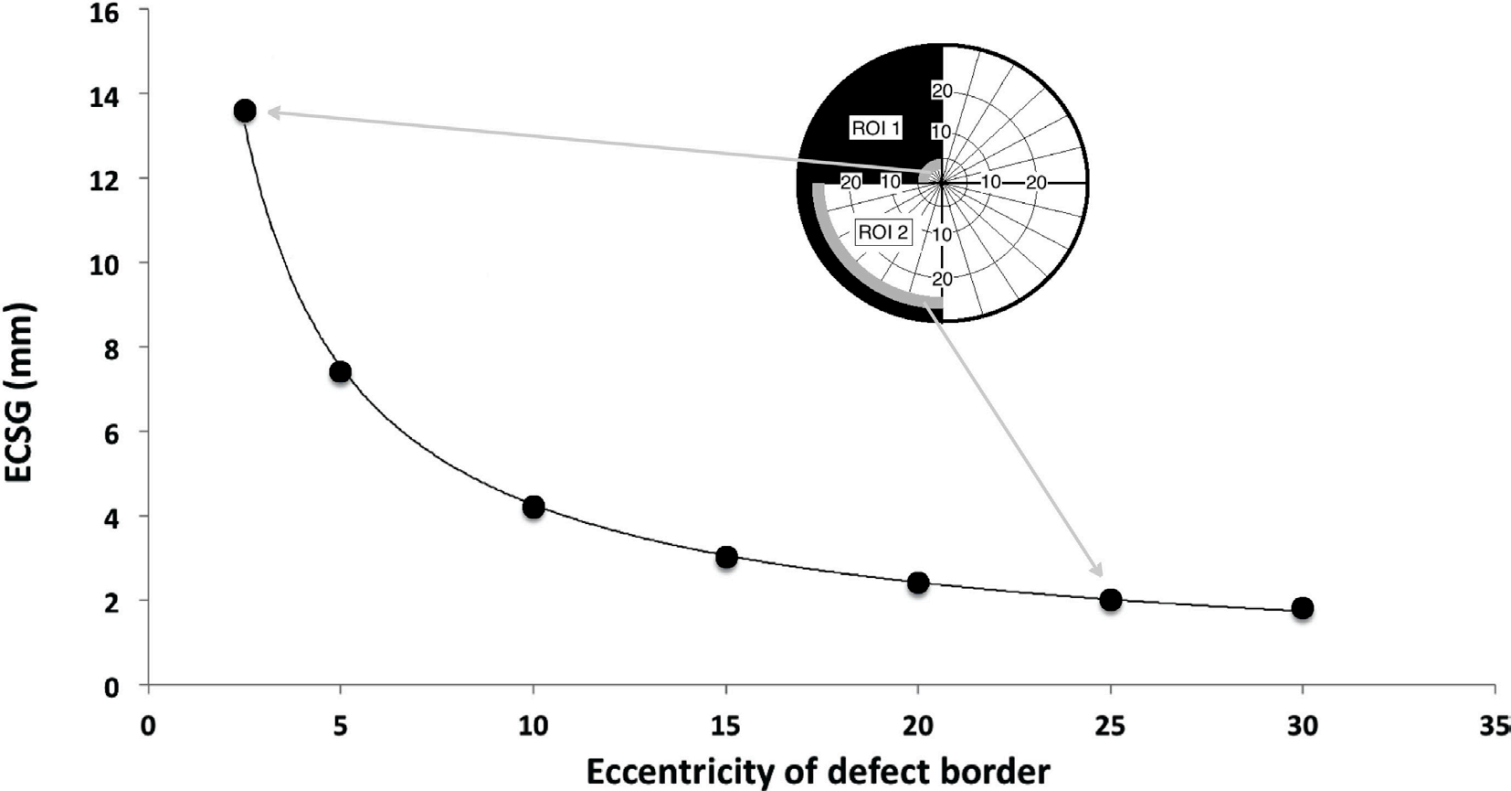
Equivalent cortical surface gain (ECSG) (in mm) for 2° of field increase across the entire hemifield, starting with a pretraining defect border at 2.5°, 5°, 10°, 15°, 20°, 25°, and 30° eccentricity. Positive ECSG indicates an increase in eccentricity of the visual field in millimeter cortex representation in V1. Insets show a 2° field increase in region of interest (ROI) 1 and ROI 2 for a defect border initially located at 2.5° and 25°, respectively. If the 2° field increase occurs in a sector of β degrees wide, numbers on the vertical axis are to be multiplied by β/180. Consequently, the contribution of the upper half of the trained hemisphere to ECSG of the depicted training result is 7.0 while the lower quadrant contributes 1.2.

Figure 3 illustrates the ECSG for a border shift of 2° across the entire border of both ROIs as a function of eccentricity. For a more detailed description of ECSG calculation, we refer to Elshout et al. (11).

Equivalent cortical surface gain values were determined after both training rounds for both the trained and untrained areas, resulting in four ECSG measurements per patient (ECSG*_k_*; *k* = 1, 2, K, 4). Thus, each separate ECSG measurement represents a visual field gain in comparison with the previous measurement. The sum of these four values determines the total ECSG (tECSG), which is a measure of the total visual field recovery. tECSG can differ considerably between patients but this measure is by itself *not* indicative of the relative contributions of spontaneous and trained recovery.

To capture the pattern of increase in the trained and untrained regions of the visual field during the two training rounds, we *normalized* each patient’s ECSG_k_ scores on that patient’s maximum of the four ECSG measurements:

(1) $${\text{nECSG}}_{k}={\text{ECSG}}_{k}/\text{Max}\left[{\text{ECSG}}_{i};i=1,2,\ldots ,4\right].$$

The *normalized* ECSG (nECSG) per subject on the one hand prevents overrating patients with huge improvements and, on the other hand, blowing up noise patterns in patients without improvement (e.g., when using tECSG ~0 for normalization).

### Follow-up Perimetry

In subacute patients, visual field recovery was retested in a follow-up session, on average 14.1 months after their training was concluded. We used this period without training to estimate the effect of “No Intervention.” During the follow-up session, we were able to apply eye tracking during Goldmann perimetry to probe potential effects of inadequate fixation. We used a perimeter-mounted eye tracker (Eyelink II, SR Research, ON, Canada) with a sample rate of 250 Hz to track the eye contralateral to the defect hemifield. The camera was attached to the base support of the chin rest using a flexible holder (see Figure 2B). Before perimetry, the system was calibrated manually, displacing the target within the perimeter as directed by the EyeLink. Then, the white size IV stimulus was shown at 10° eccentricity in horizontal and vertical directions and eye position recorded for off-line analysis.

We made three perimetry maps. First, the eye ipsilateral to the damaged field was measured without eye tracking to re-familiarize the patient with the perimetry procedure. Then, the other eye was measured twice: once with and once without eye tracking. The order of the latter two was switched for every consecutive patient, and the date on which the patient was able to visit determined this order. For every measurement, a new score sheet was used.

This procedure allowed us to estimate the magnitude of fixation errors during Goldmann perimetry that may have gone unnoticed by the spyglass method. It also allowed us to reject measurements with fixation errors in excess of 1° during the last 2 s before tapping, and to assess the effect of fixation errors on the uncorrected field map.

Finally, by comparing the follow-up field map with the posttraining map, we could probe the stability of the training effect over time. Perimetry maps were compared using the ECSG measure for the border shift between two maps (see below). Thus, we established ECSG_post-followUp_ and ECSG_followUp-followUpEL_, where _“post”_ stands for the post training visual field map without recording, _“followUp”_ stands for visual field during the follow-up without eye tracking and _“followUpEL”_ stands for visual field during the follow-up with eye tracking, the measurements without adequate fixation pruned.

Of the 17 patients that were analyzed, 14 subacute patients participated in follow-up Goldmann perimetry with eye tracking; data from one subject had to be discarded because of eye tracking failure. On average 18% of the trials per perimetry had to be discarded due to eye excursions outside the 2 deg wide fixation region. Taking for each subject the ECSG of the visual field when inadequate fixation scans were discarded, we found a mean (±SEM) difference of −0.21 ± 0.13 mm in comparison with the ESCG where inadequate fixations are included. While the mean difference between the fields without and with eye recording were as follows: ECSG_followUp-followUpEL_ = 0.17 ± 0.15 mm. Apparently, the effect of inadequate fixation on the estimation of the field border was minor in these patients.

In chronic patients, during intake also a perimetry was performed. As the intake preceded the onset of the training by about 3 months, the difference between the perimetry during intake and the baseline measurement provided the non-intervention effect on the field defect for chronic patients.

### Reading

To assess reading speed before, during and after training, we used two texts (15-point Arial, ranging from 88 to 168 words) placed on a reading stand. The patient’s head was stabilized on a chin rest 50 cm in front of the stand. We recorded eye movements with the Eyelink II eye tracker (head mounted) while patients read the texts silently. Reading speed [words per minute (WPM)] was calculated from the time between the first and the last saccade that was made during reading a text. Reading speed of the two texts was averaged. The effect of training on reading was calculated as the percentage increase in reading speed (%iWPM):

(2) $$\text{\%iWPM = }100\times \left({\text{WPM}}_{\text{post}}/{\text{WPM}}_{\text{pre}}–1\right).$$

For each training round, we used the individual data of chronic and subacute groups together to perform a two-dimensional regression between percentage increase in reading speed and ECSG_trained_ of the trained ROI and ECSG_control_ of the control ROI.

Percentage reading speed increase was collected for each epoch separately, i.e., by comparison with baseline following epoch 1, and by comparison with the intermediate measurement after epoch 2.

### Goal Attainment Scaling

Goal Attainment Scaling was applied before and after the entire training was completed. In, GAS, a number of personal and realistic (i.e., attainable) goals are set in cooperation with the experimenter (26). Goals that require major recovery of an almost complete hemianopia are not realistic because training effects usually are confined to the vicinity of the defect border. A realistic goal is a goal that “fits” the expected location and size of defect reduction. The subject’s choice of the goals ensures that the goal –when achieved- is relevant for activities of daily life (ADL). Examples of these goals are: no more/less bumping against stationary objects; no more/less walking into branches when mowing the lawn; being able to navigate a website; being able to respond quicker to approaching moving objects from peripheral visual field areas; seeing enough to complete crossword puzzles; find objects quicker; being able to read with a comfortable speed, etc.

Following the formulation of personal goals, the baseline levels of activities pertaining to certain goals were assessed before training. The baseline levels are always set at “−2.” Then, the level of a targeted goal is set at “0.” After training, the levels pertaining to the chosen goals are reassessed. These reassessed levels range from −3 to +2 (See Table 1).

**Table 1:** Goal Attainment Scaling levels.

−3	Deterioration in comparison with baseline
−2	Baseline (no change)
−1	Improvement, but less than goal level
0	Improvement to targeted goal level
+1	Improvement better than targeted goal level
+2	Improvement far better than targeted goal level

Finally, the patient assigned weights to the goals so that an order of relevance was established (least relevant = 1). When three set goals improved to the targeted level (“0”), a GAS score of 50 was reached.

After completion of the entire training, the patient and experimenter assessed the level of each goal again. Details regarding the GAS scoring method can be found in Elshout et al. (11).

### Statistical Analyses

Statistical testing was performed with SPSS version 23. 17 subacute stroke patients were included for analysis (training between 6 and 26 weeks poststroke) and 7 *chronic* stroke patients (training *at least* 9 months poststroke, on average 18 months poststroke). Repeated measures 2 × 2 factorial design ANOVAs were used to test for main and interaction effects (“trained ROI vs. control ROI” × “first training round vs. second training round”) on field change (nECSG). ANOVAs were done for each patient group separately and for both groups combined.

We evaluated reading speed increase (%iWPM) in relation to the change of the visual field (ECSG_trained_ and ECSG_control_), and we evaluated GAS (change in GAS score) in relation to the total change of the visual field (tECSG) by regression analysis.

14 Subacute patients participated in follow-up Goldmann perimetry using eye tracking, and 13 patients were included for analysis. We used Signed-Rank Tests for paired sample comparisons to test for interaction effects (training vs. no intervention; no training vs. no intervention) with respect to field change.

## Results

### Field Change: Total ECSG

**Figure 4:**
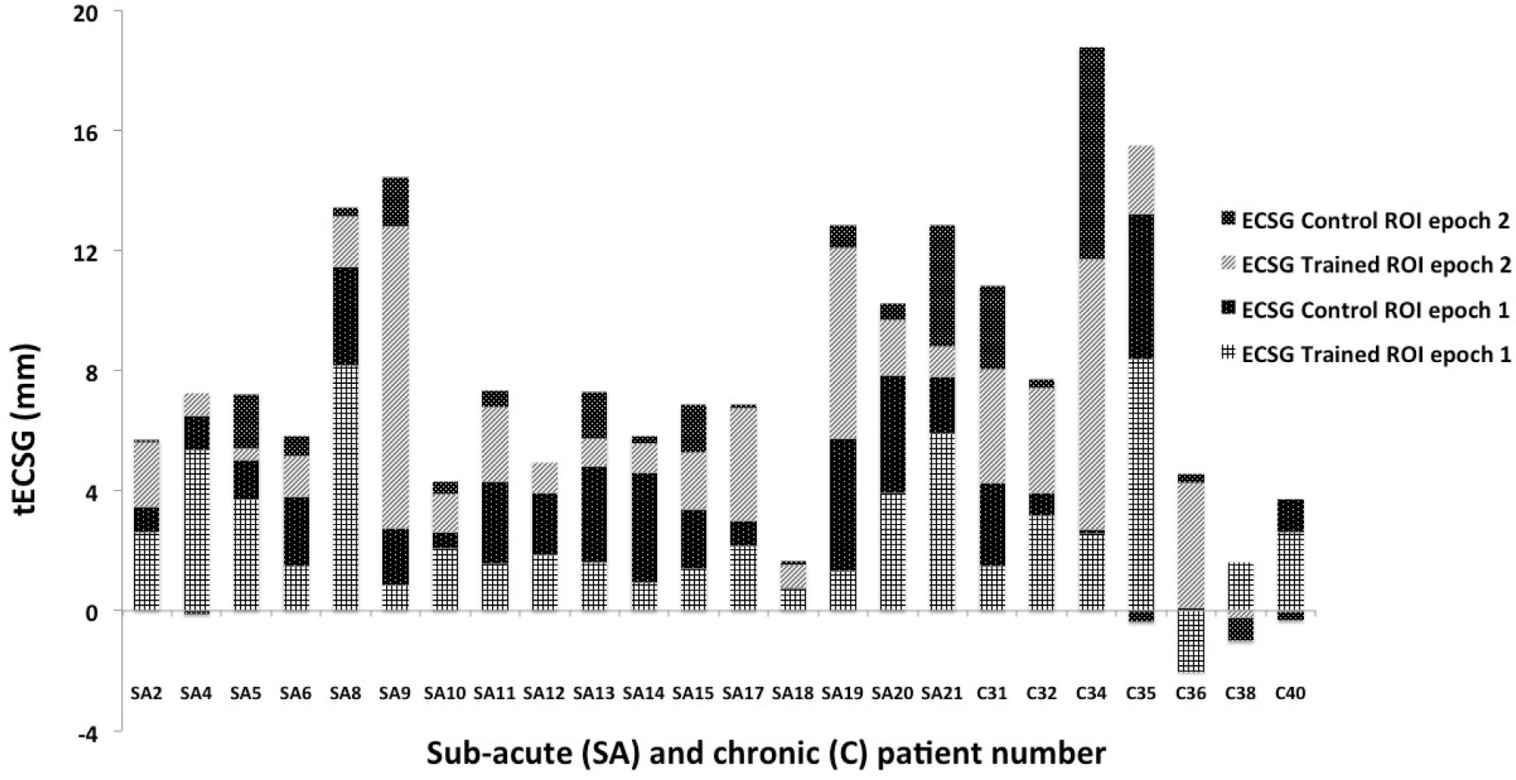
Total ECSG (tECSG) of all patients: summation of equivalent cortical surface gains (ECSGs) of both control and trained regions of interest (ROIs) across both training rounds. Note that some patients also show negative values after one (SA 4; C35 and C36) or both training epochs (C38, C40).

**Figure 5:**
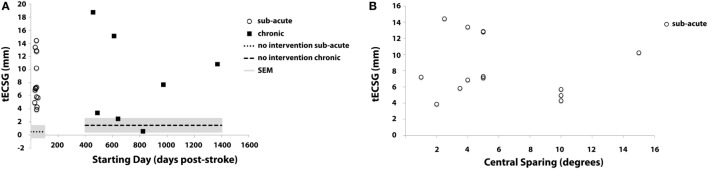
Total ECSG (tECSG) as a function of **(A)** starting day of training relative to the CVA for all patients, **(B)** macular sparing in subacute patients. The dotted line in panel **(A)** shows the average tECSG in the subacute group after a period of non-intervention (period between post measurements—follow-up). The dashed line shows the average tECSG in the chronic group after a period of non-intervention (period between intake—baseline measurement).

Total ECSG sums the contributions to defect reduction of both control and trained ROIs across both training rounds. In Figure 4, these values are shown for both the subacute and the chronic patients. tECSG varied between 1 and 18 across all chronic and subacute patients. Mean (±SEM) of tECSG for subacute and chronic patients was 7.9 (±0.9) and 8.4 (±2.6) mm, respectively. We also measured tESCG after a period of non-intervention (Figure 5A, dashed lines). For the subacute group, mean (±SEM) of tECSG [between the post-measurements and the follow-up measurements was (0.52 ± 3.18 mm)]. For the chronic patients, mean (±SEM) of tECSG (between the intake and the first measurement) was 1.5 ± 1.0 mm.

This variation appeared not related to start-time of the training following stroke (Figure 5A). In the Supplementary Material, we show the change of the visual field across the training for all analyzed subjects (Figures S1 and S2 in Supplementary Material).

We also analyzed the contributions of the trained ROI and the control ROI separately to the tECSG in the subacute group. Thus, we summed the ECSG of the trained ROI for both training rounds to obtain tECSG_trained_ and likewise we obtained tECSG_control_.

Note that these two numbers each represent the growth of the visual field in the entire defect.

Next, using the Wilcoxon Signed Rank Test, we observed that either characteristic is significantly larger than the change of the defect during the period without intervention:

tECSG_trained_ (4.76 ± 2.35 mm) is significantly larger than tECSG_follow-up_ (0.52 ± 3.18), *z* = −2.97, *p* = 0.003 and
tECSG_control_ (3.26 ± 1.63 mm) is significantly larger than tECSG_follow-up_ (0.52 ± 3.18), *z* = −2.27, *p* = 0.023.

Also, we found that tECSG_trained_ (4.76 ± 2.35 mm) was significantly larger than tECSG_control_ (3.26 ± 1.63 mm), *z* = −2.580, *p* = 0.010. When we limited this analysis to the 13 subjects for which we collected follow-up data, near significance was reached: *z* = −1.922, *p* = 0.055.

Previous work suggested that central sparing is positively correlated with spontaneous recovery (1). Macular sparing ranged from 1° to 15° for the subacute stroke patients. Indeed, a trend to increased tECGS with increased amount of central sparing is visible, but it is not significant (Figure 5B).

### Field Change: nECSG

Total ECSG (tESCG) was significantly larger than the tECSG during the periods without intervention in either patient group (dashed lines Figure 5). This confirms for our subacute patients previous observations in chronic patients (11) that during the training period a significant field reduction occurs. To discern the contributions of spontaneous recovery and training, we split the tECSG in the vector of four contributions from the two training rounds and the two trained regions. As with tECSG, nESCG appeared not related to start time of the training following stroke (slope not significant). Next, we used ANOVA’s to test for effects of training round, trained region and their interaction. We found that there was a significantly larger nECSG for the trained area than for the untrained area, *F*(1,23) = 19.2 (*p* < 0.0001, partial η^2^ = 0.46) including chronic and acute patients. This also holds separately for the subacute patients *F*(1,16) = 9.52 (*p* < 0.05, partial η^2^ = 0.37) and the chronic patients *F*(1,6) = 12.03 (*p* < 0.05, partial η^2^ = 0.67).

Which area is trained/untrained has no effect when including both patient groups *F*(1,23) = 1.79 (n.s.), subacute patients *F*(1,16) = 1,45 (n.s.), or chronic patients *F*(1,6) = 0.61 (n.s.) separately.

The interaction between trained area and training round is significant when the data of all patients are considered *F*(1,23) = 4.56 (*p* < 0.05; partial η^2^ = 0.165) or when the data of the subacute patients are considered *F*(1,16) = 10.47 (*p* = 0.005, partial η^2^ = 0.40).

**Figure 6:**
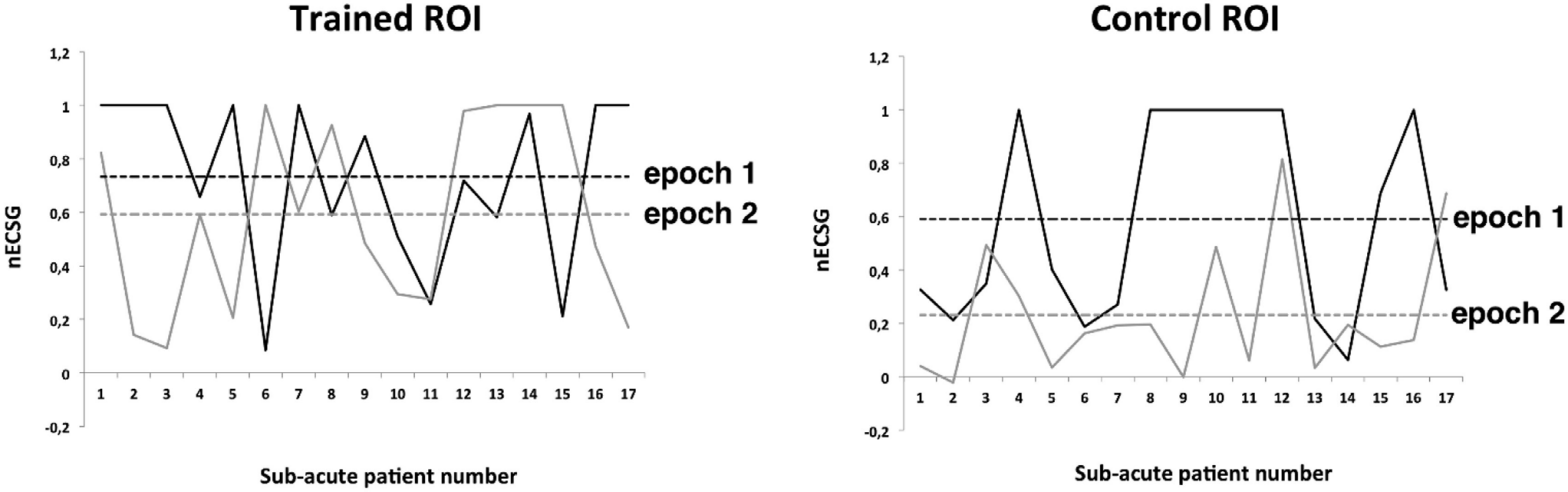
Normalized ECSG (nECSG) for both epochs (training rounds) of trained and control (non-trained) regions of interest (ROIs) of the subacute patients. Dashed lines indicate group’s average nECSG levels per training round (epoch) and region. For each panel, both parts of the defect are depicted. In epoch 1, ROI 1 was trained and ROI 2 served as control. In epoch 2, this was reversed.

In the subacute group, the effect of training decreased much more with time in the control regions (Figure 6 right) than for the trained regions (Figure 6 left), suggesting that spontaneous recovery is time dependent but trained effect is much less so in the subacute group. *Post hoc* paired samples *T*-tests confirmed this: for the trained regions, nESCG did not differ, *t*(16) = 1.039, *p* = 0.314 but for the control regions, nESCG was significantly lower following the second training round than after the first, *t*(16) = −3.63, *p* < 0.005. No such interaction is indicated in the chronic group, in which nECSG was on average three times smaller for the control ROI (0.2 ± 0.3) than for the trained ROI (0.6 ± 0.4), independent of training round.

This result made us wonder if we could estimate from the observed changes in the two ROIs for the subacute patients and the chronic patients the different contributions of training (*T*), spontaneous recovery (*S*), and spillover (*s*).

The measured values are the nESCG for the trained ROI and the nECSG for the control ROI. From these measured values we can compute *S, T*, and *s*, which stand for the (*S*)pontaneous component of the border shift, the (*T*)rained component of the border shift, and the (*s*)pillover component of the border shift. We can obtain the following equations for the contributions of *s, S*, and *T* to the training in the subacute group (note: the index “*i*” stands for the training round):

$${\text{nECSGcontrolROI}}_{i}=\;S_{i}+T_{i}.$$

In the *chronic group*, nECSG was on average three times smaller for the control ROI, therefore *s* = *T*/3.

We assume now that this ratio holds also for the subacute group irrespective of training round.

$${\text{nECSGcontrolROI}}_{i}=\;S_{i}+\;s_{i}=\;S_{i}+\;T_{i}/3.$$

Solving this simple set of equations, we arrive at

$$T_{1}=\;0.21,\;S_{1}=\;0.52,\;s_{1}=\text{ 0.07 for the first training round}$$

and

$$T_{2}=\;0.54,\;S_{2}=\;0.05,\;s_{2}=\text{ 0.18 for the second training round.}$$

Hence, we observe that the recovery by training is on average 1.5 times spontaneous recovery. As stated before spill over is about one-third of the trained recovery. Because this is also an effect of training, in our design training evoked nearly twice as much recovery as spontaneous recovery. Hence, in the subacute patients, about one-third of the total training result is due to spontaneous recovery and two-third as a result of visual training. Note that because the total ECSG was about equal in the two patient groups and because there is no spontaneous recovery in the chronic group, trained recovery was *apparently* larger in the chronic than in the subacute patients.

Interestingly, in the subacute group, nearly the same ratio between nECSGtrainedROI and nECSGcontrolROI was found in the second training epoch as in the chronic training group (Figure 6 gray dashed lines). During the first training epoch this ratio is about 1.25 (Figure 6 black dashed lines).

### Field Change: No Intervention

In-between the final training session and the follow-up measurements of the subacute patient group, there was a period without training, which we denominate “No Intervention.” The mean ECSG_post-followup_ (field change after no intervention) was 0.5 ± 0.9 mm (Figure 5A dashed line, left); at the group level, the visual field remained stable during the period between the end of the training and the follow-up measurement. Individually, fields did change. For the majority (nine subjects) unsigned ECSG_post-followup_ was less than 2 mm. In the other four subjects, ECSG_post-followup_ varied between +8 and −4. For the chronic group, ECSG_intake-baseline_ (no-intervention period between intake and the start of the training) was 1.5 ± 1.0 mm (Figure 5A dashed line, right).

### Reading Speed

We collected percentage reading speed increase for each epoch separately, i.e., in comparison with baseline after epoch 1 and in comparison with epoch 1 after epoch 2. For epoch 1, data of 21 patients were collected; for epoch 2, data of all 24 patients were available. Reading speed changes could vary considerably, ranging from decreases in reading speed by 40% to increases by 150%. The corresponding visual field change of each training round was characterized by the ECSG_t_ of the trained ROI and the ECSG_c_ of the control ROI.

We regressed %iWPM to ECSG_trained_ and ECSG_control_ during each epoch (1 or 2). We found the following regression functions:

After epoch 1, %iWPM = −0.14 + 0.070*ECSG_control_1 + 0.129*ECSG_trained_1,
After epoch 2, %iWPM = 0.034 + 0.001*ECSG_control_2 + 0.022*ECSG_trained_2,
For epoch 1, only the regression coefficient of ESCG_trained_ was significantly different from 0, *F*(2,18) = 10.59, *p* < 0.005, the regression coefficient of ESCG_control_ was not significant,
For epoch 2, the same applied: only the regression coefficient of ESCG_trained_ was significantly different from 0, *F*(2,21) = 0.98, *p* < 0.05, the regression coefficient of ESCG_control_ was not significant.

**Figure 7:**
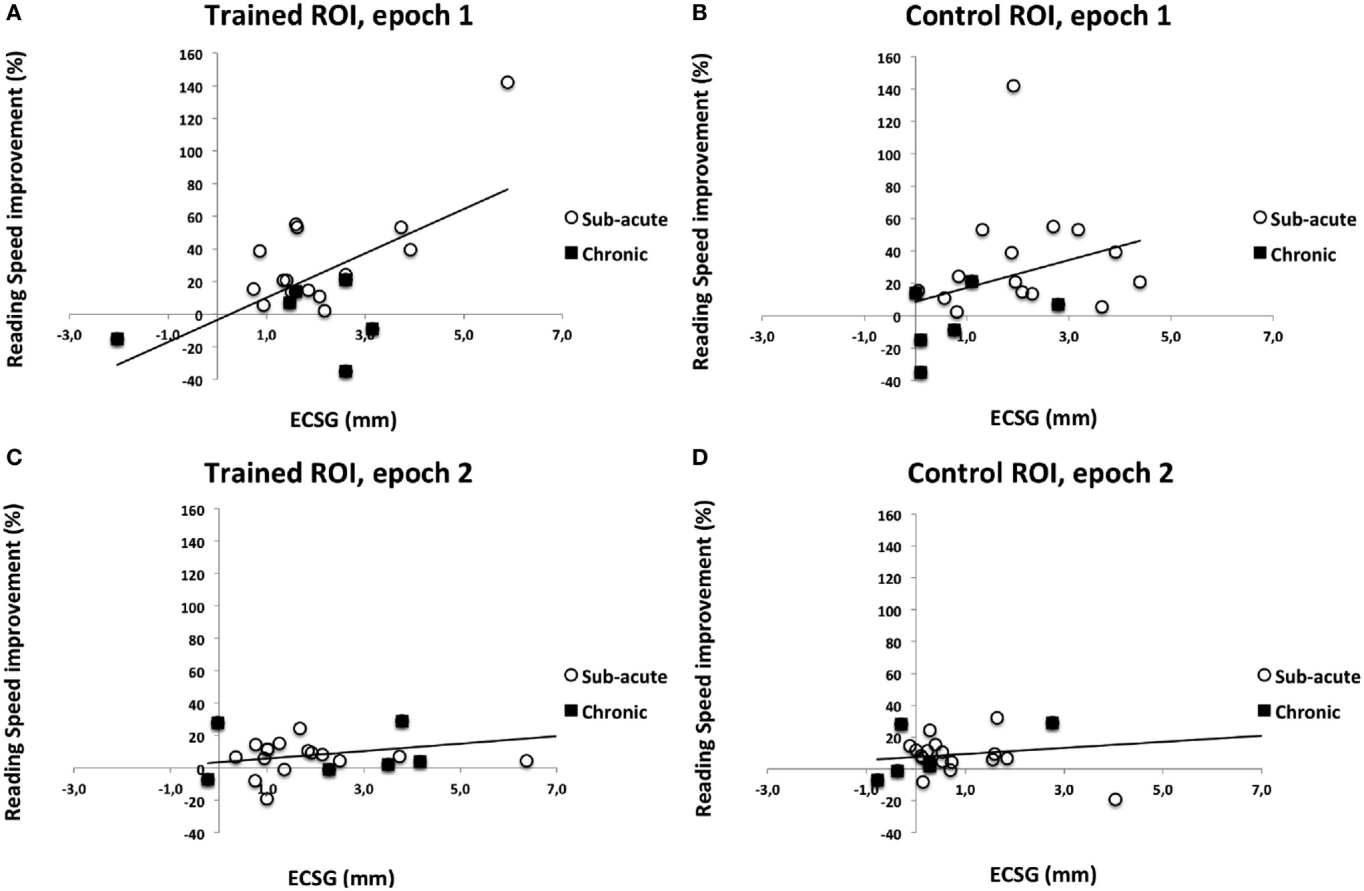
Percentage increase of reading speed as a function of equivalent cortical surface gain (ECSG) for all training rounds and regions. Data from subacute and chronic patients are combined in each panel. **(A)** Trained region during first training round; **(B)** control region during first training round; **(C)** trained region second training round; and **(D)** control region second training round.

In both epochs (Figure 7), there is significant increase of reading speed with ECSG of the *trained* ROI, but not with ECSG of the *control* ROI. So, increase of reading speed is related to training-induced recovery.

### Goal Attainment Scaling

Goal Attainment Scaling was applied before and after training. One of the chronic patients was struck by another stroke after training was completed, precluding the second GAS measurement. Thus, GAS data were obtained in only 23 patients. To the best of our knowledge, there is no proper scoring method defined for comparing more than two GAS measurements, preventing the analysis of training round effect on GAS improvement. So, our GAS scores do not permit to be associated with either ROI that was trained. Therefore, we cannot make inferences about GAS scores for the separate training epochs or ROIs.

**Figure 8:**
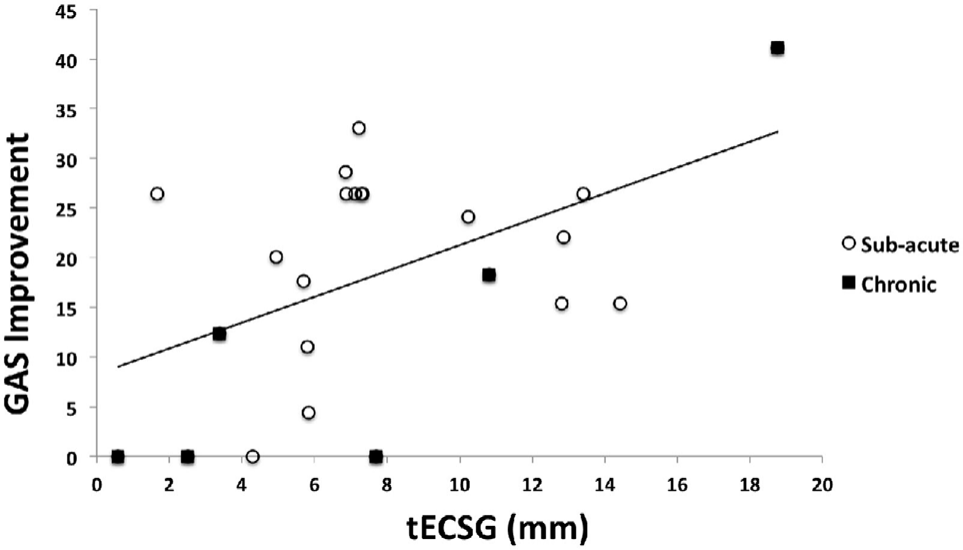
Goal Attainment Scaling (GAS) score improvement as a function of total ECSG (tECSG) for subacute and chronic patients combined (*N* = 23).

Three goals were set for most patients. For patients SA12, SA16, and SA20, two goals were set and for subject SA9, four goals were set. GAS improvements are shown in Figure 8. Initial scores ranged between 22 and 25.9. On average, a GAS improvement of 26 was needed to fully reach the set goals (GAS score 50). Clearly GAS improvement varied between no effect of training at all (GAS improvement 0) to improvement beyond the set goals (GAS improvement >26). About one-third of the patients reached their goals (7), one-third reached about halfway (7) and the rest improved little or not at all (9).

We found a significant increase of GAS with tECSG (Figure 8):

GAS = 8.3 + 1.3∗tECSG,

F(1,22)=7.02(p<0.05).

This importantly illustrates that the extent of the field enlargement is predictive of the GAS improvement.

A summary of all patient data can be found in Table S1 in Supplementary Material.

## Discussion

We report, for the first time to our knowledge, on the separate contributions of spontaneous recovery and training to visual field recovery in people with hemianopia in the subacute phase.

To test the hypothesis that training in the early phase leads to a larger defect reduction than training in the chronic phase, we must distinguish between these two components of recovery. To do so, we split the visual field defect in two equal halves that were trained in turn. The other region served as a control region to monitor spontaneous recovery and spillover from the training of the neighboring region. Our previous work on training patients in the chronic phase (11) has shown that even across hemispheres some amount of crossover training occurs. Because such crossover training effect (spillover) might be different between neighboring parts of the field defect, we applied the same training design to people with hemianopia in the chronic phase. Given that spontaneous recovery is excluded in people with hemianopia in the chronic phase, any recovery in the control region is interpreted as “spillover.”

Our results permit the following major conclusions:

(A) Training induces significant visual field recovery on top of recovery that is found in the untrained control region for the subacute group. In trained and control regions, recovery is significantly larger than recovery in a period without intervention and without spontaneous recovery. This mimics the previous observations in people with hemianopia in the chronic phase that visual training improves visual performance (5–11, 27, 28).
(B) In the subacute patient group we found a significant decline of the recovery with training round (i.e., time) in the control region but not for the trained region. In people with hemianopia in the chronic phase, spill over and training effect are not time dependent. Elshout et al. (11) found a significant interaction between trained hemifield and training order, indicating more effect on Goldmann field isopters of training the defect side first. Because the current training involved training of the defect in both training rounds, one cannot directly compare these outcomes. Yet, we note that a hint of a larger spillover effect in the first training round is apparent but not significant.
(C) By taking the nECSG scores (Figure 6), we can estimate the contributions of spill over, spontaneous recovery and training to the reduction of the field defect. We used a simple model with three independent contributions to nECSG: trained recovery (*T*), spillover of trained recovery to the control region (*s*), and spontaneous recovery (*S*). Spillover (*s*) is assumed to be the same in subacute and chronic patients. (*T*) and (*S*) come from measurements.
  Our model assumes that the trained region shows the effects of *T* + *S*, while the control region shows the effects of *s* + *S*. Also for the chronic patient group *S* = 0 (no spontaneous recovery). Note that we found nearly identical results for nECSG from the second training round for subacute patient group and both training rounds of the chronic patient group. This suggests that the rate of spontaneous recovery had nearly dropped to zero in the second training round of the subacute patients.
  The nECSG data of the chronic patient group indicate that nECSG of the control ROI was on average three times smaller than nECSG of the trained ROI. From this, we can infer the ratio between *s* and *T* in the chronic phase: *s* = *T*/3. We observed that the recovery by training is on average 1.5 times spontaneous recovery. As stated before spill over is about one-third of the trained recovery. Because this is also an effect of training, in our design training evoked nearly twice as much recovery as spontaneous recovery. Hence, in the subacute patients, about one-third of the total training result is due to spontaneous recovery and two-third as a result of visual training.
  We admit this is no more than a rough estimate of the relative contributions of the different recovery components. Nevertheless, this analysis suggests that the visual training also in the subacute phase adds substantially to spontaneous recovery, albeit not as much as in the chronic patient group. This appears to conflict with the popular view that training effects may be larger in the period directly following the stroke event because of physiological changes in the cortex directly surrounding the damaged region (13). Given that we started training about 6 weeks after the event, we believe a more definite conclusion should be postponed until our result would be confirmed when training starts in the first two weeks.
(D) Our derivation in (C) indicates that the *S* declined to 10% of the recovery rate in the first training round over a period of 8 weeks, which would suggest an exponential decay with a time constant of about three weeks for the spontaneous recovery rate. Zhang et al. (2) reported for hemianopia patients a gradual decrease in likelihood to find visual field enlargement on a second perimetry measurement depending on the time interval between the stroke and the initial measurement. He showed a drop off of this measure from about 60 to 40% when patients were first tested within 2 weeks or between 1 and 2 months after stroke. No recovery on the second visit was found if the first visit had occurred about 6 months after the event. This outcome was confirmed recently (29) in a study comparing the Humphrey visual field at 2 and 6 months after stroke, and extended by that study with the observation that recovery appeared to be most likely in the peripheral and lower field quadrants. We cannot confirm the latter conclusion from our observations, because our patient group was too small. Neither previous study provides enough detail about the extent of the recovery or the time at which it occurred to allow for a quantitative estimate of the decay of recovery rate with time. Taken together it appears that the bulk of spontaneous recovery occurs in the first three weeks after stroke although some spontaneous recovery may still be found up to about 6 months.

However, we should mention that clipping effects (i.e., when the entire border of the first trained ROI has reached 30° eccentricity at the end of the first training round) creates a bias toward a lower spontaneous recovery in the second training round. After all, the spontaneous recovery during the second training round in that region can only be “0” in our paradigm. This has happened for part of the border in SA13 and SA17 and indeed nearly completely in SA20. Thus, this effect may have biased downward our estimate of the amount of spontaneous recovery during the second training round. This may provide a partial explanation why we find such a quick decay of spontaneous recovery, quicker apparently than suggested from previous work.

So far, we have focused on the perimetric changes, but the ultimate goal of rehabilitation is an improvement of daily life measures. Previous work in chronic patients reported significant improvement following training using questionnaires (30, 31) reading speed (9, 10) and eye saccades during a driving test (28). Here, we monitored two parameters: reading speed increase and success in achieving improvement on visually affected tasks of daily living (GAS). Both measures were significantly improved following the training, but we cannot tell whether that increase was significant compared with a period without intervention. Importantly, however, we found a significant linear dependency between the growth of the visual field and the improvement on either measure. This strongly suggests that the growth of the visual field has contributed to the improvement of ADL.

Only one previous study reported GAS improvements after visual training in chronic hemianopia patients (31), but GAS improvement and field improvement appeared unrelated, possibly because goals had not been set realistically (e.g., peripheral recovery goals in hemianopia patients with a small macula sparing).

Interestingly, we found that reading speed increased proportionally to the extent of the trained improvement of the visual field, not to the extent of spontaneous recovery. An increase of the visual field may benefit reading speed because a larger horizontal extent in the direction of reading may benefit gaze orientation relative to word boundaries, while a larger horizontal extent in the opposite direction may benefit directing gaze to the beginning of the next line, and recognition of the starting letters of a word when a saccade lands in its middle. Thus we would expect that subjects with a relatively larger recovery (larger ECSG) would show a larger gain in reading speed. Indeed, this was confirmed in our group. We do not speculate why spontaneous recovery would somehow contribute less than trained field recovery to improve reading speed. Possibly the larger recovery in trained region than the control region by itself helped to lift it to significance.

We conclude then that visual training in the subacute phase promotes visual field recovery and thereby helps the patient to achieve a set of goals of daily living. The field recovery is not larger than for patients in the chronic phase, suggesting—at least for now—that early training merely benefits the patients by offering them the field enlargement earlier.

## Ethics Statement

All subjects gave written informed consent in accordance with the Declaration of Helsinki. The protocol was approved by the “ethical committee CMO Arnhem–Nijmegen.”

## Author Contributions

DB, JE, and AB contributed to experimental design. DB and AB contributed to data analysis and data interpretation. DB and JE contributed to data acquisition and data interpretation. All the authors were involved in the writing of the manuscript.

## Conflict of Interest Statement

The authors declare that the research was conducted in the absence of any commercial or financial relationships that could be construed as a potential conflict of interest. The reviewer NH and handling editor declared their shared affiliation.
