# Supplementary material for: Segregation of Spontaneous and Training Induced Recovery from Visual Field Defects in Subacute Stroke Patients
Source: Front Neurol. 2017 Dec 15;8:681. doi: 10.3389/fneur.2017.00681 (PMC5736566; doi:10.3389/fneur.2017.00681)
Supplement: Supplementary file 1 [file Table_1.DOCX]

| Patient | ECSG_t1_ | ECSG_c1_ | %rspeed_1_ | ECSG_t2_ | ECSG_c2_ | %rspeed_2_ | GAS1 | GAS2 |
| --- | --- | --- | --- | --- | --- | --- | --- | --- |
| SA2 | 2.60 | 0.85 | 24 | 2.14 | 0.10 | 8 | 23.56 | 41.19 |
| SA4 | 5.33 | 1.14 | - | 0.76, | -0.12 | 14 | 23.56 | 50.1 |
| SA5 | 3.73 | 1.30 | 53 | 0.34 | 1.84 | 7 | 23.56 | 56.61 |
| SA6 | 1.50 | 2.28 | 13 | 1.35 | 0.69 | -1 | 23.56 | 27.97 |
| SA8 | 8.16 | 3.23 | - | 1.68 | 0.28 | 24 | 23.56 | 50 |
| SA9 | 0.86 | 1.88 | 39 | 10.06 | 1.64 | 32 | 21.99 | 37.4 |
| SA10 | 2.07 | 0.56 | 11 | 1.25 | 0.40 | 15 | 23.56 | 23.56 |
| SA11 | 1.59 | 2.70 | 55 | 2.50 | 0.53 | 5 | 23.56 | 50 |
| SA12 | 1.85 | 2.09 | 14 | 1.01 | 0.00 | 12 | 25.9 | 45.99 |
| SA13 | .62 | 3.19 | 53 | .94 | 1.55 | 6 | 23.56 | 50 |
| SA14 | 0.94 | 3.64 | 6 | 1.01 | 0.23 | 11 | 23.56 | 34.58 |
| SA15 | 1.41 | 1.96 | 21 | 1.92 | 1.59 | 9 | 23.56 | 50 |
| SA17 | 2.18 | 0.81 | 2 | 3.74 | 0.13 | 7 | 23.56 | 52.2 |
| SA18 | 0.73 | 0.05 | 15 | 0.75 | 0.15 | - 8 | 23.56 | 50 |
| SA19 | 1.34 | 4.39 | 21 | 6.37 | 0.73 | 4 | 23.56 | 38.98 |
| SA20 | 3.92 | 3.92 | 39 | 1.85 | 0.54 | 10 | 25.9 | 50 |
| SA21 | 5.89 | 1.92 | 142 | 1.00 | 4.04 | -19 | 23.56 | 45.6 |
| C31 | 1.47 | 2.79 | 7 | 3.79 | 2.76 | 29 | 22.6 | 40.9 |
| C32 | 3.15 | 0.76 | -9 | 3.51 | 0.28 | 2 | 25.2 | 25.2 |
| C34 | 2.60 | 0.10 | -35 | 9.02 | 7.04 | 32 | 22.6 | 63.7 |
| C35 | 8.36 | 4.86 | - | 2.28, | -0.37 | -1 | 22.6 | - |
| C36 | -2.03 | 0.1 | -15 | 4.17 | 0.27 | 4 | 25.2 | 25.2 |
| C38 | 1.60 | 0.00 | 14 | -0.23 | -0.78 | -7 | 22.6 | 22.6 |
| C40 | 2.61 | 1.10 | 21 | -0.02 | -0.3 | 28 | 25.2 | 37.6 |

Supplementary Table 1. Patient data. ECSG of each patient per training round (index 1, 2) for the trained and control region (index t, c) and corresponding percentage increase reading speed per training round. GAS measurements were done prior to the first (GAS1) and following the second training round (GAS2).
