# Supplementary material for: Segregation of Spontaneous and Training Induced Recovery from Visual Field Defects in Subacute Stroke Patients
Source: Front Neurol. 2017 Dec 15;8:681. doi: 10.3389/fneur.2017.00681 (PMC5736566; doi:10.3389/fneur.2017.00681)
Supplement: Supplementary file 2 [file Table_2.DOCX]

| Patient | Gender | Age | CVA | Field defect |
| --- | --- | --- | --- | --- |
| SA2 | M | 49 | I | Hemianopia L |
| SA4 | M | 71 | I | Hemianopia L |
| SA5 | M | 39 | H | Paracentral Scotoma LL |
| SA6 | F | 71 | I | Quadranopia UR |
| SA8 | M | 62 | I | Hemianopia R |
| SA9 | F | 55 | I | Hemianopia L |
| SA10 | M | 62 | I | Hemianopia R |
| SA11 | F | 51 | I | Hemianopia R |
| SA12 | F | 68 | I | Scotoma UR |
| SA13 | M | 60 | I | Quadranopia UL |
| SA14 | M | 56 | I | Paracentral Scotoma UL + LL |
| SA15 | M | 47 | I | Incomplete upper altitudinal hemianopia |
| SA17 | M | 54 | I | Hemianopia R |
| SA18 | M | 28 | I | Quadranopia UR |
| SA19 | M | 67 | I | Hemianopia L |
| SA20 | M | 58 | H | Quadranopia LL |
| SA21 | F | 51 | I | Hemianopia L |
| C31 | F | 57 | H | Hemianopia R |
| C32 | M | 44 | H | Hemianopia L |
| C34 | M | 61 | H | Hemianopia L |
| C35 | M | 69 | I | Hemianopia L |
| C36 | M | 51 | I | Hemianopia L |
| C38 | F | 47 | I | Hemianopia R |
| C40 | M | 26 | CC | Hemianopia L |

### Supplementary Table 2. Patient demographics. I = ischaemic stroke; H = haemorrhagic stroke; CC = Contusio Cerebri; LL = Lower Left; UL = Upper Left; UR = Upper Right.
