# Supplementary figures and images for: Segregation of Spontaneous and Training Induced Recovery from Visual Field Defects in Subacute Stroke Patients
Source: Front Neurol. 2017 Dec 15;8:681. doi: 10.3389/fneur.2017.00681 (PMC5736566; doi:10.3389/fneur.2017.00681)

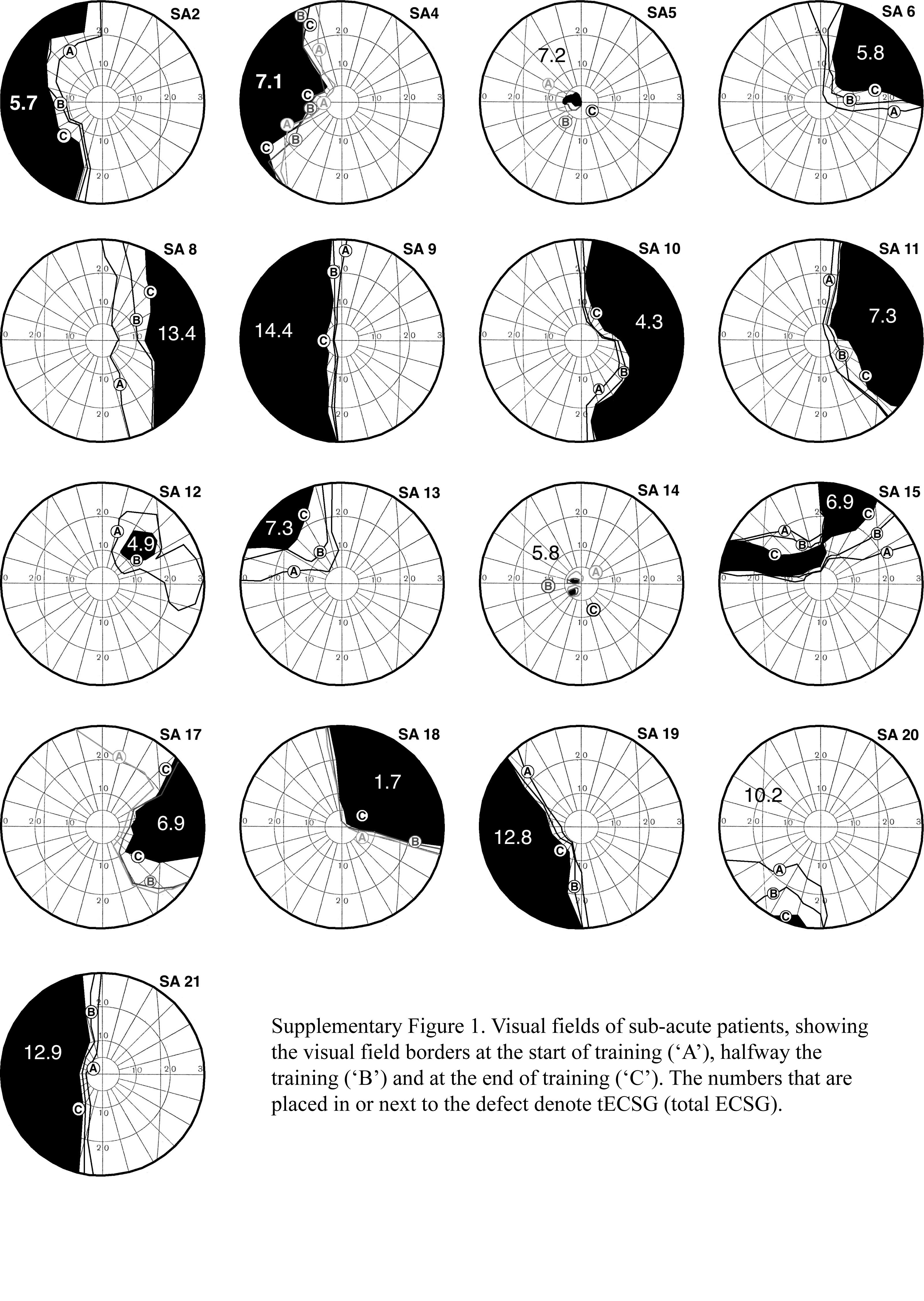

Supplement: Supplementary file 3 [file Image_1.JPEG]

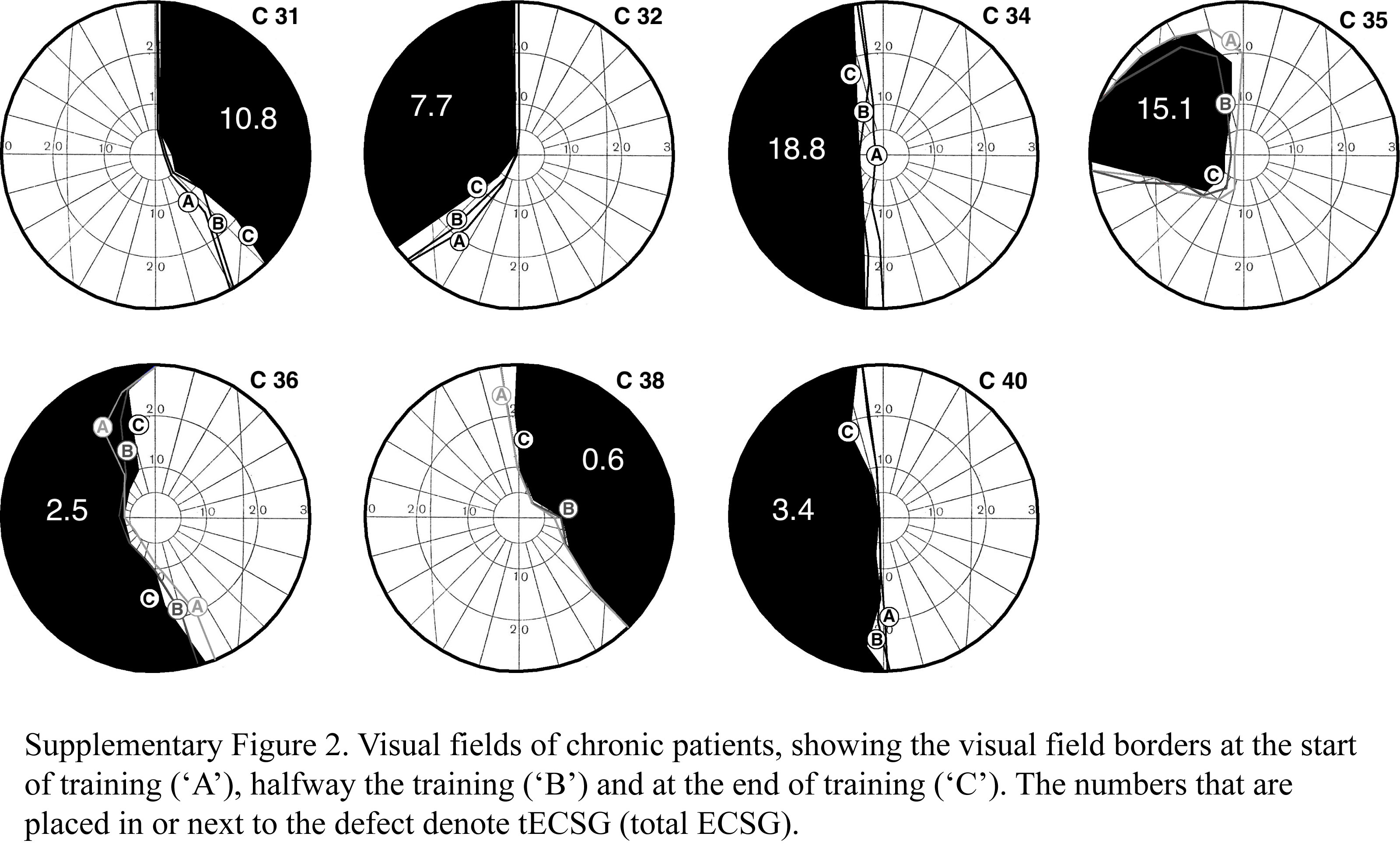

Supplement: Supplementary file 4 [file Image_2.JPEG]
